# Supplementary material for: Goal conflict in chronic pain: day reconstruction method
Source: PeerJ. 2018 Aug 15;6:e5272. doi: 10.7717/peerj.5272 (PMC6098675; doi:10.7717/peerj.5272)
Supplement: Supplemental Information 3 [file peerj-06-5272-s003.docx]

Protocol

Pain-Attention-Motivation Project 1 (PAM-I-Project)

Researchers

Claes Nathalie, MSc (KU Leuven & Ghent University)

De Paepe Annick, MSc (Ghent University)

Decoene Nele, MSc (Ghent University)

Lauwerier Emelien, PhD (UC Leuven Limburg, KU Leuven & Ghent University)

Legrain Valéry, PhD (Université catholique de Louvain)

Vlaeyen Johan, PhD (KU Leuven & Maastricht University)

Crombez Geert, PhD (Ghent University)

**1 Aim**

**1.1 goal**

The PAM-I-project investigates the role of attention and motivation upon the experience of pain and well-being in patients with fibromyalgia compared to a healthy population.

This project comprises of two independent studies, i.e. an experimental and observational study. In the experimental study, the focus lies on attentional processes. More specifically we wish to investigate the influence of chronic pain on the peripersonal representation of the body. In the observational study, we zoom in on motivational processes, more specifically on the experiences of goal conflict and its impact on pain and other patient outcomes.

**1.2 The objective of the experimental study: peripersonal space in patients with fibromyalgia (FM)**

The goal of the experimental study is to investigate the impact of chronic pain on attention towards stimuli entering the peripersonal space in patients with fibromyalgia. The goal of this study was twofold:

1. Investigating whether patients with fibromyalgia show a heightened attentional bias towards visual stimuli entering the peripersonal space compared to healthy volunteers.
2. Comparing reactions to stimuli entering the peripersonal space along a spatial continuum (from far to near space) between fibromyalgia patients and healthy control participants.

**1.3 the objective of the observational study: goal conflict in patients with fibromyalgia (FM)**

The main objective of the observational study of the PAM-I-Project is to map how often and what kind of goal conflicts individuals with fibromyalgia experience and to study its impact on the experience of pain, affect, disability, as well as to core concepts of the Fear-Avoidance model (i.e., catastrophic thinking, pain-related fear; Vlaeyen & Linton, 2000).

By using a semi-structured interviewing method and a diary assessment method, we aim at investigating:

1. Whether and to what extent patients with fibromyalgia experience goal conflict between two different activities
2. Whether there are differences in the number, type and experience of conflicts in individuals with fibromyalgia compared to a individuals not suffering from chronic pain

3. Whether the daily experience of goal conflict is associated with variations in the experience of pain, fatigue, affect or daily life

4. Whether the experience of goal conflict and its relation to the experience of pain, fatigue, affect and daily life varies according to core constructs of the Fear-Avoidance Model (e.g. pain-related fear, catastrophizing).

**2 Recruitment**

We recruited patients with fibromyalgia, and healthy controls fluent in the Dutch language, and matched for sex, age and education.

**2.1 Patients with fibromyalgia**

Patients were recruited in two ways:

1. In the period 2011-2014 posters were placed in the waiting room of the Multidisciplinary Pain Centre of Ghent University Hospital, and doctors informed patients about the possibility to participate in research. Interested patients could fill in a contact form (name, address, telephone number, e-mail) to be contacted for participation in research. A list comprising of 84 individuals with fibromyalgia was thus generated with possible candidate participants for the project. We contacted 76 of these candidates. Forty-seven individuals wished not to participate in research for numerous reasons, such as travel distance, lack of time/too busy, too much pain, other diagnosis that need treatment, etc..
2. Since august 2014, patients of the Multidisciplinary Pain Centre of Ghent University Hospital are, upon their registration at the unit, asked to fill in some questions concerning their complaints, as well as general sociodemographic information. Those data are collected via an online assessment system. After completion, a standard question is posed and asks whether patients are willing to participate in scientific research. If patients are willing to do so, their contact details are stored. This provided us with a list of possible candidate participants for the project. In total, 14 individuals with fibromyalgia left their contact information. We contacted all of these candidates. Three individuals wished not to participate in research because the travel distance was too long, or due to lack of time.

If participants left their contact information, the researchers contacted from them via telephone and arranged an individual appointment. Recruitment took place from September 2014 until March 2015. The total number of FM patients participating in the project was 40.

Inclusion criteria for patients were:

1. Being between 18-65 years of age
2. Normal or corrected-to-normal eyesight (e.g., lenses, glasses)
3. Normal or corrected-to-normal hearing (e.g., hearing aid)

Exclusion criteria were:

1. Suffering from neurological problems (e.g. epilepsy)
2. Insensitivity on the hands. This exclusion criterion was important for the experimental study, because participants had to react as quickly as possible to vibrotactile stimuli applied to the hands.
   1. If possible, participants were excluded a priori (telephone interview), if not, they were excluded during the experimental study

None of the FM patients were excluded based on these criteria.

**2.2 Healthy controls**

Since 2012, healthy candidate participants are recruited to participate in research of the Health Psychology Lab of the University of Ghent. Healthy controls are recruited in a number of different ways: via advertisements in local newspapers (e.g. Zone09), social media (Twitter, Facebook) and flyers distributed around the university campus, the local library, etc. The flyer/advertisement invited candidate-participants to leave their contact information (name, age, telephone number, e-mail address) on a website of the Research Group on Health Psychology of Ghent University (<https://www.limey.ugent.be/vrijwilligers/>). Contact information of the researchers was also placed on the flyer/advertisement, so that candidate-participants could contact the researchers in case of questions. 181 healthy candidate-participants left their contact information.

If participants left their contact information, the researchers contactedthem via telephone and arranged an individual appointment. We contacted 55 of these candidates. 14 wished not to participate in research for numerous reasons, such as lack of time/too busy, chronic pain problem or other disorder causing pain. Recruitment took place from September 2014 until March 2015. The total number of healthy controls participating in the project was 41.

Inclusion criteria for controls were:

1. Being between 18-65 years of age
2. Normal or corrected-to-normal eyesight (e.g., lenses, glasses)
3. Normal or corrected-to-normal hearing (e.g., hearing aid)

Exclusion criteria were:

1. Suffering from neurological problems (e.g. epilepsy)
2. Insensitivity on the hands. This exclusion criterion was important for the experimental study, because participants had to react as quickly as possible to vibrotactile stimuli applied to the hands.
   1. If possible, participants were excluded a priori (telephone interview), if not, they were excluded during the experimental study
3. Suffering from pain of a severe intensity (Von Korff, Keefe, & Dworkin, 1992)
   1. During the individual appointment, pain severity was assessed with the pain severity and disability scale of Von Korff (Von Korff Ormel, Keefe, & Dworkin, 1992). Controls were excluded (unbeknownst of the participant) when they were classified in category of grade III or higher, that is when their pain was at least moderately disabling.
4. Meeting the criteria for Fibromyalgia, as defined by Wolfe et al.( 2010).
   1. During the individual appointment, the Fibromyalgia diagnostic criteria were completed. If controls had a widespread pain index (WPI) ≥ 7, and a symptom severity scale score of ≥ 5, or a WPI 3-6 and SS scale score ≥ 9, they were excluded.

Three control participants were excluded for suffering from a severely intense pain (grade III, grade IV). Another control participant was excluded for meeting the diagnostic criteria for Fibromyalgia. The final sample of control participants was 37.

All participants completing the laboratory session received reimbursement for their expenses.

In Figure 1, an overview of recruitment and drop-out/exclusion is presented.

**Figure 1. Overview recruitment and drop-out.**

**b)Control participants**

**a) Patient participants**

**3 Procedure**

All participants were invited for an individual appointment at Ghent University, which took approximately 3 hours. Before the appointment, participants were requested to complete several questionnaires. Questionnaires were preferably filled in online (n=74), but paper versions were available for participants who did not have access to the internet/a computer (n=7). The individual appointment consisted of both studies: the experimental crossmodal cueing task and a semi-structured interview (one part of the observational study). Next, participants were invited to fill in a diary 14 consecutive days (second part of the observational study). In the following, the experimental and observational part will be described separately.

**3.1 Questionnaires to be filled out at home**

All participants completed the following questionnaires:

- Sociodemographic information (age, sex, socio-economic status, education level)
- Hand/Foot dominance
- Pain Catastrophizing Scale (*PCS; Crombez et al., 1998*)
- Body Vigilance Scale (*BVS; Schmidt et al., 1997*)
- Depression Anxiety and Stress scales (*DASS; Beurs, 2001*)
- Positive and Negative Affectivity Scale (*PANAS, Watson & Tellegen, 1998*)
- Spielberger Trait Anxiety Inventory (*STAI; Van der Ploeg et al., 1980*)
- The Experience of Cognitive Intrusion of Pain scale (ECIP ; Attridge, Crombez, Van Ryckeghem, Keogh, Eccleston, 2015)
- Ruminative Response Scale (RRS; *Raes & Hermans, 2007*)

Patients with fibromyalgia additionally completed the following questionnaires:

- Information about pain (location, duration, treatment,…)
- Pain Disability Index (*PDI; Pollard, 1984*)
- Pain Vigilance and Awareness Questionnaire (*PVAQ; Roelofs et al., 2003*)
- Pain Anxiety Symptoms Scale (*PASS; McCracken et al., 1992*)

**3.2 Individual Appointment**

81 participants (40 patients, 41 healthy controls) completed the experimental study and the observational interview.

**3.2.1 Information and informed consent**

Upon arrival in the laboratory, all participants were informed about the course of the project, which first comprised of some extra questionnaires, followed by an experimental crossmodal cueing task and a semi-structured interview based on the Daily Reconstruction Method (Kahneman, Krueger, Schkade, Schwarz, & Stone, 2004). During that interview, participants would receive information on a third part related to the interview, that is, a daily event sampling procedure for 14 consecutive days. The individual appointment lasted about 180 minutes.

**3.2.2 Questionnaires**

Before the start of the experimental task, we administrated the criteria for fibromyalgia (Geenen & Jacobs, 2010; based on Wolfe et al., 2010) and the pain severity and disability scale of Von Korff (Von Korff Ormel, Keefe, & Dworkin, 1992). Control participants matching the criteria for fibromyalgia or experiencing chronic pain were excluded from the analyses (see above).

**3.2.3 Study 1: peripersonal space in FM**

**A Stimuli and apparatus**

Tactile stimuli were presented by means of two resonant-type tactors (C-2 TACTOR, Engineering Acoustics, Inc., Florida, <http://www.eaiinfo.com>), consisting of moving magnet linear actuators in a housing of 3.05 cm diameter and 0.79 cm high, with a skin contactor of 0.76 cm diameter. The tactile stimuli had a frequency of 300 Hz, and a duration of 10 ms. The intensity of the tactile stimuli was determined for each participant individually by means of a random staircase procedure. 20 tactile stimuli were presented to one of the hands (i.e. the ‘reference hand’, for half of the participants this was the left hand, for the other half the right hand) (the intensity started between 0 and 0.27 Watt) and self-reports were collected on a 11-point Likert scale (0 = ‘not intense at all’ to 10 = ‘very intense’). The intensity that elicited an average rating of 7 was selected as the intensity of the tactile stimulation for the reference hand during the experiment. The perceived stimulus intensity of the other hand was matched (Weinstein, 1968). This was done by means of a double random staircase procedure, based on the ‘simple up-down method’ of Levitt (Levitt, 1971). 24 stimuli on this hand were judged relative to the stimulus on the reference hand on a 5-point Likert scale (1 = ‘more than less strong’, 2= ‘less strong’, 3= ‘equally strong’, 4= ‘stronger’, 5= ‘much stronger’). The intensity that elicited an averaged rating of 3 was used as the intensity of the stimulus on the other hand.

The visual stimuli were presented by means of fourteen green light-emitting diodes (LEDs), and one red LED for fixation.

The participants sat on a chair in a dimly illuminated, sound-attenuated room, with their head position fixed in a chin rest. The height of the chin rest was individually adapted. Participants rested their arms on the table in front of them, and placed their hands, palm downward on the table. The distance between the participants’ hands and their trunk, as well as the distance between the participants’ index fingers was 40 cm. In total 14 LEDs were positioned at different distances from the hands. 7 LEDs were positioned in the left side of space, and 7 LEDs in the right side of space. At both sides, the first LED was positioned in between thumb and index finger, the next six LEDs were positioned on a straight line, one in front of the other with 12 cm in between successive LEDs, so that the last LED was 72 cm in front of the first LED. On each trial, the LEDs on one side were successively illuminated, creating the illusion of a light coming closer towards the participant (the last LED, at 72 cm distance, was first illuminated), or going further away from the participant (the first LED, in between thumb and index finger, was first illuminated). Each LED was illuminated for 280 ms, so that the total dynamical visual stimulus had a duration of 1960 ms. A red fixation LED was positioned in between the LEDs in left and right space, 36 cm in front of the first LEDs. This fixation LED was illuminated at the beginning of each trial, and was turned off for 1s after a response was given.

**B Procedure**

The experiment started by illuminating the LEDs one by one. Participants were asked to look at the fixation LED and to indicate verbally at which side of space a light was illuminated (i.e. “left” or “right”). This was done to ensure that participants could see all the LEDs. Next, participants completed a practice phase of 14 trials, in which they had to achieve 90% correct performance in order to proceed with the experiment. All participants reached this criterion.

The procedure is based on a study of Canzoneri et al. (2012). Each trial started with the illumination of the fixation LED for 1s. Thereafter the dynamical visual stimulus started. At different temporal delays after the onset of the visual stimulus, a tactile stimulus could be presented: T1, a tactile stimulus was administered 170 ms from light onset; T2, 450 ms from light onset; T3, 730 ms from light onset; T4, 1010 ms from light onset; T5, 1290 ms from light onset; T6, 1570 ms from light onset; T7, 1850 ms from light onset. This was true both for the approaching and the receding light. In this way, the light was perceived at different locations with respect to the body at the moment the tactile stimulation occurred. For example, when the light was approaching it appeared close at high temporal delays. Conversely, when the light was receding, it appeared close at low temporal delays (see Figure).

The experiment consisted of 8 blocks of 56 trials each. The trials were created by crossing the moving direction of visual stimulus (approaching vs. receding) with the congruency of the visual and tactile stimulus (congruent vs. incongruent), the side at which the visual stimulus was presented (left/right side of space) and the 7 different temporal delays (T1 - T7). 1/8 of the trials (i.e. 7 trials) per block were randomly assigned as catch trials, in which no tactile stimulus was presented.

Participants were instructed to keep their gaze on the fixation LED during the whole block. They were asked to respond as fast and accurately as possible to the occurrence of a tactile stimulus. Responses were given by means of two foot pedals, one positioned beneath the toes, and one beneath their heel. Participants were instructed to keep the foot pedals depressed during the experiment, and to lift either their toes or their heel to respond. Participants were informed that the visual stimulus was unpredictive for the position of the subsequent tactile target. The experiment took on average 75 minutes to complete.


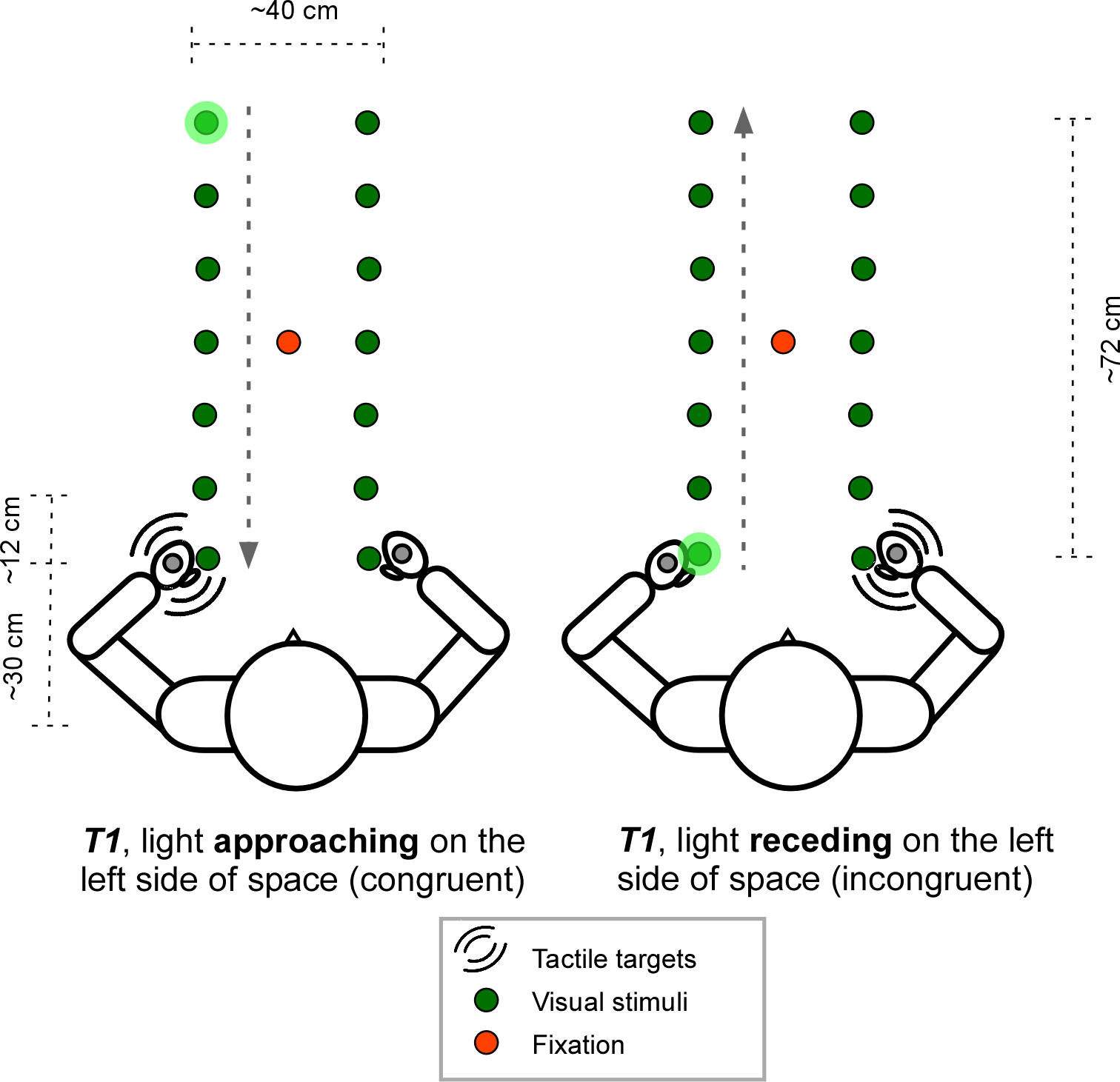


Figure. Experimental set-up. At the left side of the figure, a light is approaching the participant at the left side of space. At T1 (170 ms from light onset) the participant gets a tactile stimuli applied to the left hand (congruent to the side of space where the light is presented). At that time, the light is at 72 cm from the participant’s hand. At the right side of the figure, a similar situation is depicted, however now the light is receding from the participant’s hand, so that the light is in between the thumb and the index finger at the time of stimulation. Moreover, now the right hand is stimulated (incongruent to the side of space where the light is presented).

**C Post-experiment questionnaire**

After the experiment participants were asked to complete a questionnaire containing questions about the experiment and two more general questions concerning how participants tolerate the proximity of stimuli/other people when they are in pain (see Appendix 1).

**3.2.4 Study 2: Goal conflict in FM**

At the start of the interview, the interviewer (female)^[[1]](#footnote-1)^ informed the participant about the goal and procedure of the Day Reconstruction Method (for the full instructions in Dutch, see Appendix 2).

**A Reconstruction of previous day**

Before reconstructing their previous day, the researcher asked the following questions on a Likert scale from ‘0’ (not at all) to ’10’ (very much):

- How much pain are you currently experiencing?
- How tired are you at this moment?

. Next, the date and day of yesterday were filled in. Subsequently, the experimenter asked at what time the participant woke in the morning and at what time they went to sleep. Next, participants could start reconstructing their day from waking until going to sleep in key words.

More specifically, participants were told that they would reconstruct the activities they did during the previous day (yesterday). It was stressed that the interviewer would not criticize the participants and/or the activities mentioned, and that by no means details should be shared with anybody. Rather, a description of the activity in a couple key words would be sufficient. The interviewer emphasized that if participants were uncomfortable disclosing some information, they could indicate this to the interviewer and describe it in a way they were comfortable with. The participants were given the time they needed to think about and reflect about their previous day. The experimenter explained that the day would be divided in three parts, that is “morning” (from waking until noon), “afternoon” (noon until about 18:00), and “evening” (from about 18:00 until going to bed). There was room for about 10 activities per part, but it was possible to mention less or even more activities. Activities reported could be from 15 minutes up to 2 hours. The interviewer explained that when during the interview sensitive topics would surface, the participant could take a break or this can be briefly discussed, and that in any case, participants would receive contact information of the researcher responsible as well as professional workers (e.g., medical doctors, psychologist, etc.). Lastly, participants could look back on their previous day to check whether they wished to alter, add or delete activities.

**B Conflict mapping**

Consecutively, the interviewer informed participants that in the following, the focus is not on the activities per se, but on the experience of conflict between activities. The experimenter explains what conflict in the context of this experiment means:

“*Conflict means* ***experiencing some indecisiveness or doubt about which activity to pursue.*** *For example a student can doubt whether to study a chapter for his exam, or to go out for drinks with friends; a parent can doubt between going to the swimming class of his/her daughter, or doing grocery shopping; another person can experience doubt whether to read the newspaper or repairing the leaky faucet; another person can doubt between resting to reduce pain, or going for dinner with friends.
Furthermore,* ***it is important that this indecisiveness or doubt about which activity you wish to pursue persist for some time, that is, that you do not immediately can make a decision on what to do. Also, it is important that the activities sufficiently differ from each other.*** *Sufficiently differ means that both activities are not based on the same act. For example: when you are sitting in a restaurant doubting whether you would eat steak or shrimps does not fit this description of conflict, since eating shrimp and eating steak are based on the same act, that is, eating. However, if you are in that same restaurant doubting whether to eat a dessert or going back to work, it fits our description of conflict. I also wish to emphasize that we do not mean social conflict, that is, having a fight or an argument with another person or persons, as is usual when we are discussing conflict.”*

The interviewer checked whether the participant understood the definition of conflict, and asked him/her to give a (general) example of a conflict that fits this description. If the example was incorrect, the experimenter explained why and clarified the description. Another example was asked. When the provided example was correct, the interviewer asked to think back of yesterday again, and indicate whether they had experienced a conflict. The definition as described above (without examples) was placed in front of the participant as a reminder. Each conflict received a conflict code. The number of conflicts was recorded for all participants.

Per conflict, a form was completed, consisting of the following elements:

- Time of conflict (Time conflict)
  - in hh:mm
- Description in key words of the first activity (Activity 1)
  - Qualitative description
- Description in key words of the second activity (Activity 2)
  - Qualtitative description
- With whom was the participant (Who)
  - Qualitative description
- Where the participant was at the time of conflict (Where)
  - Qualitative description
- Whether the conflict was caused or introduced by someone else, and if yes, by whom? (Social influence)
  - Dichotomous answer
  - If yes, qualitative description
- Reasons of conflict/doubt (Reasons)
  - Qualitative description
- Duration of conflict/doubt (Duration)
  - In seconds
- Decision which activity to pursue:
  - Answer options: 1 of both activities, 2 activities (mostly sequential), no activities of the conflict

When the participant has described all his/her conflicts, the interviewer summarizes and asks whether the participant wishes to change, delete or add something.

**C Goal categories**

Next, participants were informed that per activity per conflict, the immediately underlying goal will be classified. To this purpose, several goal categories were formulated (Chulef, read, & Walsh, 2001). The researcher explains the goal categories one by one to the participant. The different goal categories are:

1. **Interpersonal/Social**: The goal is to maintain or improve contact or relationships with other people (eg., going out with friends)
2. **Intrapersonal**: the goal is to maintain or improve personal qualities or personal growth (e.g., being friendly)
3. **Work/Education**: the goal is related to work and/or educational purposes, and is aimed at the personal (academic) carrier (e.g., following classes, meeting deadlines,…)
4. **Household**: the goal is to pursue household activities or chores, and is aimed at maintenance or improving your household (e.g., having a clean house)
5. **Pleasure/Leisure**: The goal is to relax or to enjoy yourself, mostly the goal is to pursue activities that are aimed at things you do in your spare time (e.g., hobbies)
6. **Financial**: The goal is to maintain or improve your financial status, freedom, independence, security or stability
7. **General Physical and Mental Health**: the goal is to maintain or improve your general physical and/or mental health, e.g. eating healthy food, stress reduction; with the exception of the goal to avoid, reduce or control pain.
8. **Pain control, avoidance and/or reduction**: the goal is to control, avoid or reduce pain, e.g., resting, avoiding movements, taking medication
9. **Other**: if the goal does not fit in one of the other categories, this category can be selected. A description of the ‘other’ category can be provided.

The interviewer first checked if all goal categories are clear for the participant, and next are asked to classify each of the activities of the conflicts they reported in one of the abovementioned categories. The interviewer emphasized that only one goal per activity could be selected. When multiple goals were applicable, the participant was instructed to choose the most important goal. A list of the goal categories is placed in front of the participant as a reminder. The interviewer first provided an example how to classify the goals of the activities:

*“Imagine sitting in a restaurant and doubting between staying for a chat with your friend, or going back to work. You may want to chatt with your friend because you want to maintain the relationship with him/her. This can be placed in the category “social/interpersonal”. The goals immediately underlying going back to work can be because you just wish to do the work you are meant to do; this can be classified in the category “work/education”. However, it is also possible that you wish to go back to work because you want to be a professional and hard-working person, which can be classified in the category “intrapersonal”. Another goal you can have, is to obtain a financial bonus; this can be placed in the category “financial”. Since multiple goals are present, you have to pick the one that was most applicable in that situation, e.g., “work”.*

Participants could ask for additional clarification if necessary. Next, participants classified all their conflicts.

**D Conflict assessment**

Subsequently, the interviewer requested the participant to completeseveral questions regarding the conflict. If the participant mentioned three or less than three conflicts, the questions were answered for all conflicts. When participants reported more than three conflicts, three of the conflicts were selected at random using an a priori randomization scheme (see Appendix 2).

The questions were, all answered using a Likert scale going from 0 (not at all) to 6 (very much) :

- How strongly did you experience this conflict? (conflict strength)
- To what extent did you worry during this conflict? (worry)
- To what extent did you worry about pain during this conflict?(worry about pain)
- To what extent did you feel stressed during this conflict? (stress)
- To what extent did you need support during this conflict? (support)
- How difficult was it to solve this conflict? (conflict solution)
- How satisfied were you with the solution of this conflict? (solution satisfaction)
- To what extent did you feel […] during the conflict?:
  - Happy
  - Enthusiastic
  - Relaxed
  - Sad
  - Nervous
  - Irritated
  - Angry
  - Afraid
  - Powerless
  - Frustrated
  - Helpless

**E Assessment of previous day**

Lastly, participants completed a questionnaire regarding their day of yesterday. The questions were:

- How typical was yesterday compared to other days the past month?
  - Scale from 0 (not typical at all) to 10 (very typical)
- Compared to other days the past month, yesterday was…
  - 0 – much worse, to 10 – much better
- How satisfied were you with your day of yesterday?
  - 0 – not satisfied at all, to 10 – very satisfied
- To what extent did you do what you planned to do yesterday?
  - 0 – not at all, to 10 – very much
- To what extent did you pursue valuable activities yesterday?
  - 0 – not at all, to 10 – very much
- How tired were you yesterday throughout the day, on average?
  - 0 – not tired; 10 – very tired
- How much pain did you experience yesterday throughout the day, on average?
  - 0 – no pain; 10 – worst pain imaginable
- To what extent did fatigue hinder you yesterday while performing your activities?
  - 0 – not at all, to 10 – very much
- To what extent did pain hinder you yesterday while performing your activities?
  - 0 – not at all, to 10 – very much
- To what extent did you think about fatigue yesterday?
  - 0 – not at all; to 10 – very much
- To what extent did you think about pain yesterday?
  - 0 – not at all; to 10 – very much
- To what extent did you feel (on a scale from 0 – not at all; to 6 – very much)…yesterday
  - Happy
  - Enthusiastic
  - Relaxed
  - Sad
  - Nervous
  - Irritated
  - Angry
  - Afraid
  - Powerless
  - Frustrated
  - Helpless
  - Confident
  - Loved
  - Alert
  - Optimistic
  - Restless
  - Steadfast
  - Energetic

Additionally, participants were asked how motivated they were the day of the interview to participate in the interview on a scale from 0 to 10.

**3.3 Event-sampling procedure (Diary assessment)**

At the end of the interview, the interviewer explained the event-sampling procedure (daily assessment). All participants were requested to keep a paper “diary” for 14 consecutive days in which they kept track of the conflicts they experienced during each day. The interviewer instructed participants to make a note in their diary as soon as they experienced a conflict that fit with the definition described above. Per conflict, participants were requested to indicate: the time of the conflict, the activities involved in the conflict, where they were and who was with them when they were experiencing the conflict. The interviewer showed an example of a diary and gave a step-by-step explanation. On the first page of the diary, the definition of conflict was again described, so participants could consult this at any time. Similarly, contact information of the researchers involved were placed in the diary, so that participants could contact them in case of unclarities. At the end of the day, preferably right before going to bed, for each of the 14 days, participants were requested to fill in an online questionnaire about their conflicts. In case participants did not have access to a computer and/or internet, it was possible to fill in these questions on paper. In the latter case, participants were provided with prepaid envelopes to send the filled-in questionnaire back to the researchers as soon as possible. Participants were sent an automatic text message about 19:00 pm to remind them of filling in the questionnaire.

In the questionnaire, participants indicated the number of days they had been filling in the questionnaire (the first day is 1, the last day 14), the day and date, as well as the number of conflicts experienced. Next, participants are requested to describe and classify the goals underlying each of the activities involved in the conflicts. To do so, participants were first provided with an explanation of the categories (see above), as well as an example how to fill in the questionnaire. Next, participants were requested to answer some questions regarding the conflicts they experienced (in general, on average):

- On average, how long did you doubt or were you indecisive today during a conflict experience? (duration, in seconds)
- On average, did you, on a scale from 0 (not at all) to 6 (very much)
  - Worry?
  - Worry about pain?
  - Felt stressed?
  - Needed support?
- How difficult was it today on average to solve your conflicts?
  - On a scale from 0 (not at all) to 6 (very much)
- How satisfied are you with the solution(s) of your conflict(s) today?
  - On a scale from 0 (not at all) to 6 (very much)

If participants did not experience conflicts during that day, they could select the option “not applicable”.

Furthermore, questions on their day in general were completed, independent whether goal conflicts were experienced:

- How typical was today compared to other days the past month?
  - Scale from 0 (not typical at all) to 10 (very typical)
- Compared to other days the past month, today was…
  - 0 – much worse, to 10 – much better
- How satisfied were you with your day of today?
  - 0 – not satisfied at all, to 10 – very satisfied
- To what extent did you do what you planned to do today?
  - 0 – not at all, to 10 – very much
- To what extent did you pursue valuable activities today?
  - 0 – not at all, to 10 – very much
- How tired were you today throughout the day, on average?
  - 0 – not tired; 10 – very tired
- How much pain did you experience today throughout the day, on average?
  - 0 – no pain; 10 – worst pain imaginable
- To what extent did fatigue hinder you today while performing your activities?
  - 0 – not at all, to 10 – very much
- To what extent did pain hinder you today while performing your activities?
  - 0 – not at all, to 10 – very much
- To what extent did you think about fatigue today?
  - 0 – not at all; to 10 – very much
- To what extent did you think about pain today?
  - 0 – not at all; to 10 – very much
- To what extent did you feel (on a scale from 0 – not at all; to 6 – very much)…today
  - Happy
  - Enthusiastic
  - Relaxed
  - Sad
  - Nervous
  - Irritated
  - Angry
  - Afraid
  - Powerless
  - Frustrated
  - Helpless
  - Confident
  - Loved
  - Alert
  - Optimistic
  - Restless
  - Steadfast
  - Energetic

The researchers called the participants around the 7^th^ day of filling in the diary, to check whether they applied the correct definition of “conflict” (by providing an example of conflict) and which strategy they used to fill in the diary and questionnaire. It was also an opportunity for participants to ask questions.

Similarly, participants were contacted again after having completed the diary (after the 14^th^ day) to check if the period they filled in the questionnaire was normal/abnormal and what impact keeping the diary and filling in the questionnaire had on their experiences.

Participants completing the questionnaire at least 10 consecutive days and sending the diary back to the responsible researchers with the provided prepaid envelope, received a reward. Amongst participants completing the questionnaire and diary 14 consecutive days, 2 vouchers of 50 euros (to spend in FNAC, a store with CDs, DVDs, software, and electronics) were raffled.

For the patient group (N = 40), 11 did not start the diary or did not complete at last 10 consecutive days. Reasons for not starting/completing the diary were: no time or considering it too much work. The remaining sample for analysis was 29 (see Figure 3).

For the control group (N = 41), 4 did not start the diary or did not complete more than 10 days. Similar to patients, most often control participants did not wish to complete the diary because of lack of time. The 4 participants meeting the exclusion criteria, were excluded. Additionally, 2 participants did not comply to the researcher’s instructions, and were excluded from data-analyses as well. In total, the control sample for the diary data comprised of 31 controls (see Figure 3).

**Figure 3. Overview of diary inclusion.**

**4 Remarks**

The experimental study of the PAM-I-Project is part of the doctoral project of Annick De Paepe, who is supported by the Research Foundation Flanders (FWO, Belgium, FWO12/ASP/080). The project is entitled “Where is my pain? A neurocognitive investigation into the spatial perception of pain”. Both observational components are part of the doctoral project of Nathalie Claes, which is supported by the research grant ‘‘Pain-Related Fear in Context: The Effects of Concomitant Non-pain Goals and Goal Conflicts on Fear Responding in the Context of Pain’’ funded by the Research Foundation–Flanders (Fonds Wetenschappelijk Onderzoek [FWO] Vlaanderen), Belgium, granted to G.C. and J.W.S.V. (grant ID: G091812N).

**5 Contact**

**UGent***Department of Experimental-Clinical and Health Psychology
Health Psychology Lab*
Henri Dunantlaan 2, B9000 Ghent, Belgium
E-mail: [geert.crombez@ugent.be](mailto:geert.crombez@ugent.be)
Tel: +32(0)9 264 64 61

**KU Leuven**
*Research unit Behavior, Health and Psychopathology
Research Group on Health Psychology*
Tiensestraat 102 box 3726, B3000 Leuven, Belgium
E-mail: [johan.vlaeyen@ppw.kuleuven.be](mailto:johan.vlaeyen@ppw.kuleuven.be)
Tel: +32(0)16 32 59 15

**Appendix 1 – Post-experiment questionnaire**

PPNR:

Gelieve volgende vragen in te vullen door het gepaste cijfer te omcirkelen

1. **In welke mate heb je je ingespannen voor de taak?**

Helemaal niet Heel erg

0 1 2 3 4 5 6 7 8 9 10

1. **In welke mate kon je je concentreren op de taak?**

Helemaal niet Heel erg

0 1 2 3 4 5 6 7 8 9 10

1. **In welke mate heb je aandacht geschonken aan de visuele stimuli?**

Helemaal niet Heel erg

0 1 2 3 4 5 6 7 8 9 10

1. **In welke mate heb je aandacht geschonken aan de tactiele stimuli?**

Helemaal niet Heel erg

0 1 2 3 4 5 6 7 8 9 10

1. **Hoe intens vond je de visuele stimuli?**

Helemaal niet Heel erg

0 1 2 3 4 5 6 7 8 9 10

1. **Hoe intens vond je de tactiele stimuli?**

Helemaal niet Heel erg

0 1 2 3 4 5 6 7 8 9 10

1. **In welke mate heb je de positie van de lichtjes bewust gebruikt om te beslissen welke hand gestimuleerd werd?**

Helemaal niet Heel erg

0 1 2 3 4 5 6 7 8 9 10

1. **In welke mate vond je de lichtjes bedreigend als ze dichterbij kwamen?**

Helemaal niet Heel erg

0 1 2 3 4 5 6 7 8 9 10

1. **In welke mate vond je de lichtjes bedreigend als ze verder weg gingen?**

Helemaal niet Heel erg

0 1 2 3 4 5 6 7 8 9 10

1. **Hoe angstig/gespannen was je tijdens het experiment?**

Helemaal niet Heel erg

0 1 2 3 4 5 6 7 8 9 10

In welke mate zijn de volgende stellingen op u van toepassing?

1. **Als ik pijn heb, duld ik geen andere mensen dichtbij mij.**

Helemaal niet Heel erg

0 1 2 3 4 5 6 7 8 9 10

1. **Als ik pijn heb, lijken alle stimuli dichtbij mij veel intenser.**

Helemaal niet Heel erg

0 1 2 3 4 5 6 7 8 9 10

**Appendix 2 - Interview instructions**

**DAILY RECONSTRUCTION METHOD: PROTOCOL**

**DEEL 1. DAGRECONSTRUCTIE**

**Instructies voor onderzoeker**

*We gaan proberen het verloop van jouw dag gisteren te doorlopen en reconstrueren, zodanig dat we een goed beeld hebben op de activiteiten die je gisteren ondernomen hebt. Vervolgens gaan we enkele episodes, zoals we de activiteiten zullen noemen, een voor een in detail bekijken en zal ik je enkele vragen stellen. We proberen dus vooral een zicht te krijgen op het dagverloop, en we vellen dus geen oordeel over de verschillende activiteiten die jij onderneemt. Indien je je minder comfortabel voelt met het delen van informatie rond wat je gisteren gedaan hebt, kan je dat steeds aangeven, of het zo omschrijven op een manier waar jij je wel comfortabel bij voelt. Ook is het niet nodig om details van de verschillende activiteiten die je ondernomen uit de doeken te doen.*

*We zouden een zicht willen krijgen op wat je gedaan hebt en hoe je je voelde. Omdat veel mensen nogal wat moeite hebben om zich te herinneren wat ze gedaan hebben en wat ze ervaren hebben, gaan we stap voor stap de dag doorlopen.*

*Beschouw je dag van gisteren als een soort van continue serie van episodes, zoals scènes uit een film, waarin je een specifieke activiteit hebt ondernomen. Het is belangrijk dat je ongeveer aangeeft wanneer de activiteit begon, en hoelang de activiteit duurde. De episodes die mensen opnoemen, duren meestal 15 minuten tot een tweetal uur. Meestal begint of eindigt een episode wanneer je naar een andere locatie gaat, wanneer je een nieuwe activiteit start, of wanneer je met een nieuwe persoon een gesprek aangaat. We verwachten wel dat je een activiteit voldoende specifiek omschrijft. We bedoelen dus dat je niet louter beschrijft dat je gewerkt hebt, maar dat je ook beschrijft wat je gedaan hebt, zoals bijvoorbeeld een presentatie voorbereid. Het is tevens ook niet de bedoeling voor de indeling in episodes dat je heel specifiek beschrijft wat je gedaan hebt, zoals bijvoorbeeld beschrijven dat je eerst de lay-out hebt opgesteld, vervolgens je tekst hebt opgesteld, vervolgens je lijnen hebt ingeoefend, etc. Ik zal je steeds begeleiden bij het goed en duidelijk omschrijven van je episode.*

*Om een goed overzicht te behouden op jouw episodes of activiteiten, gaan we werken met deze tabel* [DOCUMENT DAGSTRUCTUUR]*. Je ziet een aantal vakjes staan bij voormiddag, namiddag en avond. De bedoeling is dat we in deze tabel bij benadering het tijdstip gaan schrijven waarop elke episode begon en eindigde (eerste kolom), en in de tweede kolom je activiteit beschrijven met een kort woord of een korte naam of zin die je helpt deze te herinneren (bijvoorbeeld, “pendelen naar het werk”, of “lunch met X”). Je wordt gevraagd om episodes te omschrijven voor drie verschillende ‘dagdelen’: ochtend (van het wakker worden tot omstreeks de middag), namiddag (van rond de middag tot ongeveer 18:00), en avond (van rond 18:00 tot wanneer je naar bed ging). Er is ruimte om 10 episodes in te vullen per dagdeel, maar misschien heb je er niet zoveel nodig. We gaan dan ook niet alle vakjes hoeven in te vullen, maar zoals gezegd is het wel zo dat we vragen je dag op te delen in voldoende verschillende episodes die meestal variëren van 15 minuten tot 2 uur.*

*Zo gaan we concreet te werk: we gaan je dag episode per episode mondeling overlopen. Ik zal helpen om deze over te nemen in de tabel en de episodes kort te omschrijven. Zo behoud je een goed overzicht over je dag. Als je hiermee klaar bent, zullen we samen de tabel nog eens overlopen, zodat je eventuele fouten kan corrigeren. Het is niet de bedoeling dat we in detail noteren wat jij gedaan hebt, maar dat we louter in een aantal kernwoorden samenvatten wat de activiteit omvat. Je mag dat doen op een niveau waar jij je comfortabel bij voelt. Wij vellen geen oordeel over de activiteiten die je ondernomen hebt, dat gaat voorbij aan het doel van deze studie.*

*Ik wil graag nog één iets aanhalen. Het kan zijn dat het interview gevoeligheden bij je oproept. Indien dit het geval is, dan kunnen we zeker eventjes de tijd nemen om te pauzeren, of kunnen we het rustiger aan doen. Ik moet me echter ook aan een strikt tijdsschema houden. Dit maakt dat we geen ruimte hebben om van het interview af te wijken. Moest het zo blijken dat het interview zaken bij je oproept die je graag nog met een professionele werker zou willen bespreken, dan kan dit uiteraard zeker. Ik geef je aan het eind van onze afspraak een lijst mee met contactgegevens.*

*Als alles duidelijk is, kunnen we van start gaan met het reconstrueren van je dag. Geef gerust aan wanneer je klaar bent om van start te gaan.*

***Als niet klaar****: Geef je me even aan wat je precies onduidelijk vond? Dan kunnen we het nog eens samen overlopen.*

***Als klaar****: We kunnen starten met eerst een aantal algemene vragen over gisteren te beantwoorden. Nu we dit hebben ingevuld, gaan we verder met het beschrijven van je dag. Laten we starten met het moment waarop je bent opgestaan. Kan je me vertellen wat je toen hebt gedaan?*

laat patiënt mondeling dagdelen overlopen, waarna proefleider dit overneemt op het formulier DAGSTRUCTUUR.

Als de volledige dag overlopen is, vervolg met:

*Nu zou ik je willen vragen om even terug te blikken op jouw tijdslijn/tabel. Zijn er episodes die je zou willen veranderen, of misschien opsplitsen in meerdere delen? Of wil je nog ergens iets aan toevoegen?*

**Aandachtspunten**

Zorg voor een voldoende niveau van abstractie bij het omschrijven van de activiteit/episode. Laat patiënten zelf hun kernwoorden formuleren.

Maak gebruik van volgende vragen om de activiteit duidelijk omschreven te krijgen:

- Wat precies bedoel je met …?
- Kan je …. wat specifieker omschrijven?
- Waaruit bestond deze activiteit?
- Waar was je op dat moment?
- Waren er andere mensen bij jou? Wat deden zij?
- Wanneer ben je precies met deze taak gestart?
- Hoe lang duurde deze activiteit ongeveer?

Parafraseer en vat samen:

- Dus, als ik het goed begrijp ….
- Begrijp ik het goed als ik zeg dat…
- Met andere woorden…

Om tot een kernwoord te komen:

- Hoe zou je deze episode in één woord samenvatten?
- Hoe zou je deze episode noemen?
- Wat was de basisactiviteit in deze episode?

**DAGSTRUCTUUR (deel 1)**

**Hoeveel pijn hebt u op dit moment? 0 1 2 3 4 5 6 7 8 9 10**

**Hoe vermoeid bent u op dit moment? 0 1 2 3 4 5 6 7 8 9 10**

**Wat was de datum van gisteren?**

____/_____/_______ (dd/mm/jjjj)

**Welke dag was het gisteren?** (omcirkel)

MAANDAG DINSDAG WOENSDAG DONDERDAG VRIJDAG ZATERDAG ZONDAG

**Hoe laat ben je gisteren opgestaan? ____u____**

**Hoe laat ben je gisteren gaan slapen? ____u____**

| **OCHTEND** | | |
| --- | --- | --- |
| Tijdstip | Activiteit | Conflict |
| 1ochtend |  |  |
| 2ochtend |  |  |
| 3ochtend |  |  |
| 4ochtend |  |  |
| 5ochtend |  |  |
| 6ochtend |  |  |
| 7ochtend |  |  |
| 8ochtend |  |  |
| 9ochtend |  |  |
| 10ochtend |  |  |

| **MIDDAG** | | |
| --- | --- | --- |
| Tijdstip | Activiteit | Conflict |
| 1middag |  |  |
| 2middag |  |  |
| 3middag |  |  |
| 4middag |  |  |
| 5middag |  |  |
| 6middag |  |  |
| 7middag |  |  |
| 8middag |  |  |
| 9middag |  |  |
| 10middag |  |  |

| **AVOND** | | |
| --- | --- | --- |
| Tijdstip | Activiteit | Conflict |
| 1avond |  |  |
| 2avond |  |  |
| 3avond |  |  |
| 4avond |  |  |
| 5avond |  |  |
| 6avond |  |  |
| 7avond |  |  |
| 8avond |  |  |
| 9avond |  |  |
| 10avond |  |  |

**DEEL 2. CONFLICTMAPPING**

**Instructies voor onderzoeker**

*Nu gaan we een stapje verder in het bekijken van je dag. We zijn immers niet specifiek geïnteresseerd in welke activiteiten je onderneemt, maar eerder in de mate dat je conflict ervaart tussen activiteiten die je wil ondernemen. Dat klinkt nu waarschijnlijk wel wat vaag, maar ik zal proberen alles duidelijk uit te leggen. Indien iets niet duidelijk is voor jou, aarzel dan zeker niet om me verduidelijking te vragen.*

*Dus, zoals ik net zei, zijn we geïnteresseerd in conflict-ervaringen.* ***Met conflict bedoelen we dat er een zekere mate van besluiteloosheid of twijfel optreedt over of je nu de ene dan wel de andere activiteit zou gaan doen.*** *Bijvoorbeeld, iemand kan twijfelen tussen: studeer ik dit hoofdstuk, of ga ik op stap met vrienden. Een ander kan twijfelen tussen, ga ik kijken naar de zwemles van mijn dochter, of doe ik boodschappen. Of, lees ik de krant, of repareer ik de lekkende kraan. Nog iemand anders twijfelt: Zal ik gaan rusten om mijn pijn te doen minderen, of zal ik meegaan naar het etentje met vrienden? Is alles tot zover duidelijk?*

***Belangrijk is dat deze besluiteloosheid over welke activiteit je wil gaan doen even aanhoudt, en dat je niet onmiddellijk tot een beslissing komt. Daarnaast is het ook belangrijk dat het gaat om twee activiteiten die voldoende verschillen van elkaar.*** *Met ‘voldoende verschillen’ bedoelen we dat de activiteiten niet op eenzelfde handeling berusten. Ik geef even een voorbeeld: stel, je zit in een restaurant en je kan niet kiezen of je nu steak of mosselen zou eten. Je zou dat kunnen beschouwen als een conflict, maar het eten van steak en mosselen komt neer op eenzelfde activiteit: eten, en valt dus niet onder onze definitie. Stel nu voordat je in dat restaurant bent met een vriend(in) en je twijfelt of je nog even blijft om een babbeltje te slaan met die vriend(in), of terug naar het werk zal vertrekken om je werk verder te zetten. Uiteindelijk kies je er voor om toch nog even te blijven zitten. Dit laatste is een voorbeeld van een conflict dat onder onze definitie valt. Is alles duidelijk? [beantwoord vragen]. Ik zou hierbij ook nog eens extra willen benadrukken dat het niet om een sociaal conflict gaat, zoals gebruikelijk wanneer we over conflict spreken.*

Als **begrepen**: *Zou je mij eens een ander, algemeen voorbeeld kunnen geven van een conflict dat onder onze definitie valt?* Corrigeer het voorbeeld indien nodig.

Als **niet begrepen**: *Wat is er precies onduidelijk voor jou?* Verduidelijk waar nodig. Wanneer alles duidelijk is, vervolg met voorbeeld.

*We gaan nu terug naar jouw dag gisteren. Kan je terugdenken aan gisteren en aangeven of je momenten hebt gehad waarop je conflict hebt ervaren volgens onze definitie? Voor alle duidelijkheid leg ik hier even de* **[DEFINITIE VAN CONFLICT*]*** *zoals we het daarnet hebben overlopen. Zo kan je er steeds naar terugkijken. Kan je even vertellen over jouw conflictmomenten gisteren?*

Laat patiënt mondeling dag en mogelijke conflictmomenten overlopen, proefleider neemt dit over in de tabel [CONFLICTTABEL] Elk conflict krijgt een unieke code.

Wanneer de patiënt klaar is met het rapporteren van conflicten, zeg dan:

*Ok, we gaan even terugkijken naar de conflicten die je hebt vermeld. Het zijn er X, waarvan X ’s morgens, X in de namiddag, en X ’s avonds. Zijn dit al jouw ervaren conflictmomenten? Wil je er nog een aan toevoegen? Is het ok op deze manier?.*

*Neem gerust nog eens een kijkje naar de tabel. Zou je graag nog iets toevoegen? Is het ok zo?*

**Aandachtspunten**

Leg het blad met de **definitie** van wat een conflict is goed centraal in het zicht van de patiënt, zodanig dat hij/zij hier steeds naar kan teruggrijpen indien nodig.

Zorg ervoor dat patiënten hun dag en mogelijke conflicten overlopen en laat ze hier **uitvoerig over vertellen**. Probeer de **essentie** eruit te halen van als ze een mogelijk conflict vermelden.

- Dus tussen welke activiteiten deed dit conflict zich voor?
- Wanneer deed dit conflict zich precies voor?
- Hoe lang duurde dit conflict precies?

Check even bij de patiënt of je het conflict **juist hebt begrepen en zo mag overnemen** in de tabel.

- Dus, als ik het goed begrijp deed het conflict zich voor tussen …. en …
- Mag ik het conflict dan als volgt omschrijven…
- Is het goed als ik het conflict dan als volgt overneem…

Let erop enkel conflicten over te nemen die **matchen** met de definitie zoals besproken. Wanneer een patiënt een conflict vermeldt dat niet strookt met de definitie, moedig hem/haar dan aan door te zeggen: “*Dat is prima, daar heb je dus een onwennigheid ervaren. Nu, dat is niet echt een conflict zoals wij het daarnet hebben overlopen. Ga gerust door met het beschrijven van je momenten en probeer er even goed op te letten dat je een conflict vermeldt die strookt met onze definitie”. Wat precies bedoel je met …?*

Let erop dat een patiënt niet teveel afwijkt van het doel van je bevraging. Zo kan iemand heel erg komen vast te zitten in een ‘existentieel’ conflict tussen leven met pijn en diens ideale situatie. Zo kan iemand, bijvoorbeeld, omschrijven dat pijn in de weg stond om terug te gaan werken. Probeer iemand dan terug te brengen tot de taak door hem/haar te zeggen: “*Het lijkt me inderdaad dat pijn en alles wat u ervoor moet doen in de weg staat van andere belangrijke dingen in uw leven. Waar we specifiek in geïnteresseerd zijn nu is het conflict dat u ervaart tussen bepaalde activiteiten die u gisteren ondernam. Kan u even terugdenken aan de definitie zoals we hebben besproken, en vertellen over de momenten waarop u mogelijks een conflict heeft ervaren gisteren?”*.

Wanneer een patiënt niet onmiddellijk begint te vertellen over zijn/haar mogelijk conflictmomenten, probeer hem/haar dan als volgt aan te moedigen:

- *Denk even terug aan je dag, aan wat je hebt gedaan, en vertel maar of je doorheen de dag hebt getwijfeld om de ene dan wel de andere activiteit te gaan doen*
- *Denk maar rustig na, en zeg gerust wat in je opkomt*
- *Heeft u ergens doorheen de dag getwijfeld over wat u zou gaan doen? Denk maar rustig na*

Het doel bestaat er in voor alle elementen van het conflict een duidelijk beeld te hebben. Het is mogelijk dat deelnemers zelf al bepaalde aspecten aanhalen, maar andere niet. Hieronder worden enkele tips opgelijst om elk van de elementen te bevragen.

**Tijdstip:**

- *Wanneer vond dit conflict precies plaats?*
- *Hoe laat was het ongeveer?*

**Activiteiten:**

- *En tussen welke twee activiteiten twijfelde je?*
- *Hoe zou je deze activiteiten omschrijven?*
- *Hoe zou je de activiteit in 1 kernwoord omschrijven?*

**Met wie was je:**

- *Was er iemand bij je? Zo ja, wie dan?*

**Waar was je:**

- *Waar bevond je je op dat moment?*
- *Waar was je toen je het conflict ervoer?*

**Werd conflict geïntroduceerd door iemand anders:**

- dit wordt doorgaans duidelijk wanneer mensen vertellen over hun conflict. Als het niet duidelijk is, geef dan een kadering van wat er bedoeld wordt: *Je haalt aan dat ….[samenvatting conflict]*. *Heeft [persoon] jou gevraagd om [activiteit] te doen, of was het jouw idee om deze activiteit te ondernemen?*
- *Heeft iemand anders het conflict veroorzaakt?*
- Parafraseren: *Dus als ik het goed begrijp…*

**Redenen van conflict/twijfel:**

- *Waarom twijfelde je tussen deze activiteiten?*
- *Wat droeg ertoe bij dat je twijfelde tussen deze activiteiten?*
- Waarom was dit een conflict?
- *Wat maakte dit een conflict?*
- *Wat is de onderliggende reden van waarom dit voor jou als een conflict aanvoelde?*

**Duur conflict:**

- *Hoe lang heb je ongeveer getwijfeld?*

**Beslissing:**

- *Wat heb je uiteindelijk beslist te doen?*
- *Heb je op een later tijdstip de andere activiteiten/beide activiteiten nog ondernomen?*

| **CONFLICTCODE**: | |
| --- | --- |
| **Tijdstip** |  |
| **Activiteit 1** |  |
| **Doel activiteit 1:**  Interpersoonlijk/sociaal  Intrapersoonlijk  Werk/opleiding  Huishoudelijk  Plezier/ontspanning  Financieel  Algemene fysieke en mentale gezondheid  Pijncontrole, -vermijding, en/of -vermindering Andere, namelijk_______________ | |
| **Activiteit 2** |  |
| **Doel activiteit 2:**  Interpersoonlijk/sociaal  Intrapersoonlijk  Werk/opleiding  Huishoudelijk  Plezier/ontspanning  Financieel  Algemene fysieke en mentale gezondheid  Pijncontrole, -vermijding, en/of -vermindering Andere, namelijk_______________ | |
| Met **wie** was je? |  |
| **Waar** was je? |  |
| Werd conflict **geïntroduceerd/veroorzaakt** door **iemand anders**? | Ja / Nee  Als ja, door wie? |
| **Redenen** van conflict/twijfel?  [Waarom was dit een conflict?] |  |
| **Duur** conflict (hoelang)? |  |
| **Beslissing**  (1 activiteiten, beide activiteiten, andere activiteit?) |  |
| **Opmerkingen**/aanvullingen |  |

Een **conflict** is het ervaren van **besluiteloosheid of twijfel** over **of je nu de ene dan wel de andere activiteit zou gaan doen**. Deze besluiteloosheid **houdt even aan**, en **je komt niet onmiddellijk tot een beslissing** over wat je zou gaan doen. De **activiteiten** waartussen je twijfelt **verschillen op voldoende wijze van elkaar**.

**DEEL 3. DOELCATEGORIEËN**

*Nu ga ik je vragen om de conflicten in verschillende categorieën te verdelen. Meer bepaald is het de bedoeling dat je voor beide activiteiten betrokken in het conflict gaat aangeven wat het achterliggende doel was, en dat je dus per activiteit aangeeft in welke categorie dat doel het best past.*

Neem de **lijst [DOELCATEGORIEËN]** bij de hand. Overloop elk van de categorieën. Vraag aan patiënten of de categorieën duidelijk zijn. Verduidelijk dat indien het doel is om pijn te vermijden/controleren/… men niet de categorie “gezondheid” moet selecteren, maar de categorie “pijn”, hoewel deze elementen ook zouden kunnen passen in de categorie “gezondheid”.

*De bedoeling is nu dat we voor elk van je conflicten classificeren op welk, onmiddellijk achterliggend doel elk van de betrokken activiteiten gericht was. Het is de bedoeling om steeds slechts één categorie per doel/activiteit te selecteren. Indien je vind dat er meer dan 1 categorie van toepassing is, gelieve dan de meest toepasselijke categorie te selecteren. Indien geen van de categorieën van toepassing zijn volgens jou, kan je de optie ‘andere’ selecteren en daar uitleggen waar het volgens jou het best in past. Ik geef even een voorbeeld. Stel dat Mieke twijfelde tussen babbelen met een vriendin en terug aan het werk gaan. Mieke wilde blijven babbelen om de relatie met haar vriendin te onderhouden, wat een sociaal doel is volgens onze classificering. Terug aan het werk gaan is voor Mieke belangrijk omdat ze hard wil werken. Mieke twijfelt een beetje waarin ze deze activiteit moet classificeren: werk/opleiding, financieel, gezien hard werk zou kunnen resulteren in een bonus, of intrapersoonlijk, omdat Mieke zichzelf graag wil ontplooien als een hardwerkend en professioneel persoon. Mieke kiest echter om het te classificeren onder ‘werk/opleiding’, gezien dat voor haar het meest van toepassing was.
Kan je dit volgen? Is het duidelijk?*

*Prima, de bedoeling is dus eigenlijk dat we dit voor elk van jouw conflicten herhalen. We gaan beginnen met het eerste conflict. Doe maar.*

Leg de **definities** voor de patiënt en laat patiënten elk conflict onderbrengen in de voor hem/haar best passende categorie. De proefleider neemt dit over in de voorziene ruimte in de **CONFLICTTABEL**.

Indien nodig, kan de proefleider gerichte vragen stellen om de patiënt te begeleiden in het kiezen van een categorie.

- *Vertel maar, tussen welke categorieën twijfel je?*
- *Kan je me uitleggen waarom je het in deze categorie zou plaatsen?*
- *Welke categorie is voor jou het meest belangrijk?*
- *Hoe zou je het omschrijven als je niet aan de categorieën gebonden was?*
- *Aan welke categorie dacht je het eerst?*

**DOELCATEGORIEËN**

| **Doelcategorie** | **Omschrijving** |
| --- | --- |
| Interpersoonlijk/Sociaal | De activiteit is gericht op het onderhouden of verbeteren van (intieme) contacten met andere mensen/derden (bv. uitgaan met vrienden) |
| Intrapersoonlijk | De activiteit is gericht op het onderhouden of verbeteren van jouw persoonlijke kwaliteiten of ontwikkeling (bv. vriendelijk zijn) |
| Werk of opleiding | De activiteit heeft te maken met het werk en/of de opleiding en is dus gericht op je (academische) carrière (bv. vergaderingen, les volgen) |
| Huishoudelijk | De activiteit bestaat uit het ondernemen van huishoudelijke taken of klusjes, en is dus gericht op het onderhouden van je huishouden (bv. schoonmaken) |
| Plezier/ontspanning | De activiteit is gericht op ontspanning of genieten; doorgaans betreft dit activiteiten die je in je vrije tijd doet (bv. hobby’s, entertainment) |
| Financieel | De activiteit is gericht op het onderhouden of verbeteren van je financiële vrijheid, onafhankelijkheid, zekerheid of stabiliteit |
| Algemene, fysieke en mentale gezondheid | De activiteit bestaat uit het behouden of bevorderen van je algemene fysieke en/of mentale gezondheid bv. gezond eten, ontspanningsoefeningen om stress te reduceren  m.u.v. activiteiten gericht op vermijden, verminderen of controleren van pijn |
| Pijncontrole, -vermijding, en/of -vermindering | De activiteit is gericht op het vermijden, verminderen of controleren van je pijn (bv., medicatie nemen, rusten, vermijden van bewegingen, warm bad nemen, enz.) |
| Andere | Indien het doel van de activiteit niet past in een van bovenstaande categorieën |

**DEEL 4. CONFLICT ASSESSMENT**

*In het volgende gedeelte gaan we wat dieper ingaan op de conflicten die je ervaren hebt. Graag zou ik samen nog een laatste maal door enkele van deze conflicten gaan, en vragen dat jij enkele vragen beantwoordt over elk van jouw ervaren conflicten* ***[INTERVIEW – beoordelen conflicten]****.*

Indien er maximaal drie conflicten werden gerapporteerd, worden alle conflicten beoordeeld. Indien er meer dan drie conflicten werden gerapporteerd, kiest de proefleider ad random drie conflicten die de patiënt heeft aangegeven, aan de hand van het RANDOMISATIESCHEMA. Bij elk conflict worden de vragen horende bij de conflictbeoordeling overlopen. Laat patiënten zelf de waarden aanduiden op papier. Altijd wordt begonnen met de conflictcode bovenaan de conflictbeoordeling te noteren. Leg de tabel met de conflictbeschrijvingen naast de deelnemer. Wijs de patiënt af en toe op het te beoordelen conflict doorheen het doorlopen van de vragen. Indien nodig, kan verdere mondelinge toelichting worden gegeven bij de te beoordelen vragen.

**RANDOMISATIESCHEMA**

|  | Te beoordelen conflicten | | | | | | | | | |
| --- | --- | --- | --- | --- | --- | --- | --- | --- | --- | --- |
| Aantal gerapporteerde conflicten | C1 | C2 | C3 | C4 | C5 | C6 | C7 | C8 | C9 | C10 |
| 4 | x |  | x | x |  |  |  |  |  |  |
| 5 | x |  | x |  | x |  |  |  |  |  |
| 6 |  | x |  | x |  | x |  |  |  |  |
| 7 | x |  |  | x |  |  | x |  |  |  |
| 8 | x |  | x |  |  | x |  |  |  |  |
| 9 |  | x |  |  | x |  |  |  | x |  |
| ≥ 10 |  |  | x |  |  | x |  | x |  |  |

|  | Te beoordelen conflicten | | | | | | | | | |
| --- | --- | --- | --- | --- | --- | --- | --- | --- | --- | --- |
| Aantal gerapporteerde conflicten | C1 | C2 | C3 | C4 | C5 | C6 | C7 | C8 | C9 | C10 |
| 4 |  | x | x | x |  |  |  |  |  |  |
| 5 |  | x | x |  | x |  |  |  |  |  |
| 6 | x |  | x |  |  | x |  |  |  |  |
| 7 | x |  |  |  | x |  | x |  |  |  |
| 8 |  | x |  | x |  |  |  | x |  |  |
| 9 | x |  |  | x | x |  | x |  |  |  |
| ≥ 10 |  |  |  |  |  | x |  |  | x | x |

|  | Te beoordelen conflicten | | | | | | | | | |
| --- | --- | --- | --- | --- | --- | --- | --- | --- | --- | --- |
| Aantal gerapporteerde conflicten | C1 | C2 | C3 | C4 | C5 | C6 | C7 | C8 | C9 | C10 |
| 4 | x | x |  | x |  |  |  |  |  |  |
| 5 |  | x |  | x | x |  |  |  |  |  |
| 6 |  |  | x |  | x | x |  |  |  |  |
| 7 |  |  | x | x | x |  |  |  |  |  |
| 8 | x |  |  |  |  |  | x | x |  |  |
| 9 |  |  | x |  |  | x |  |  | x |  |
| ≥ 10 | x |  |  |  |  | x | x |  |  |  |

|  | Te beoordelen conflicten | | | | | | | | | |
| --- | --- | --- | --- | --- | --- | --- | --- | --- | --- | --- |
| Aantal gerapporteerde conflicten | C1 | C2 | C3 | C4 | C5 | C6 | C7 | C8 | C9 | C10 |
| 4 | x | x | x |  |  |  |  |  |  |  |
| 5 |  |  | x | x | x |  |  |  |  |  |
| 6 |  | x | x |  | x |  |  |  |  |  |
| 7 |  | x |  | x |  | x |  |  |  |  |
| 8 |  |  |  | x | x |  |  | x |  |  |
| 9 | x |  |  |  |  | x | x |  |  |  |
| ≥ 10 |  |  |  |  |  | x |  |  | x | x |

|  | Te beoordelen conflicten | | | | | | | | | |
| --- | --- | --- | --- | --- | --- | --- | --- | --- | --- | --- |
| Aantal gerapporteerde conflicten | C1 | C2 | C3 | C4 | C5 | C6 | C7 | C8 | C9 | C10 |
| 4 |  | x | x | x |  |  |  |  |  |  |
| 5 | x | x |  | x |  |  |  |  |  |  |
| 6 |  | x |  |  | x | x |  |  |  |  |
| 7 | x |  |  | x |  |  | x |  |  |  |
| 8 |  |  |  |  |  | x | x | x |  |  |
| 9 |  |  | x |  | x |  |  |  | x |  |
| ≥ 10 |  |  | x |  | x |  | x |  |  |  |

|  | Te beoordelen conflicten | | | | | | | | | |
| --- | --- | --- | --- | --- | --- | --- | --- | --- | --- | --- |
| Aantal gerapporteerde conflicten | C1 | C2 | C3 | C4 | C5 | C6 | C7 | C8 | C9 | C10 |
| 4 | x |  | x | x |  |  |  |  |  |  |
| 5 | x | x |  |  | x |  |  |  |  |  |
| 6 |  | x | x |  |  | x |  |  |  |  |
| 7 | x |  | x |  | x |  |  |  |  |  |
| 8 |  |  | x | x | x |  |  |  |  |  |
| 9 | x |  |  |  | x |  |  |  | x |  |
| ≥ 10 |  | x |  |  |  |  |  | x |  | x |

|  | Te beoordelen conflicten | | | | | | | | | |
| --- | --- | --- | --- | --- | --- | --- | --- | --- | --- | --- |
| Aantal gerapporteerde conflicten | C1 | C2 | C3 | C4 | C5 | C6 | C7 | C8 | C9 | C10 |
| 4 | x | x |  | x |  |  |  |  |  |  |
| 5 |  | x | x | x |  |  |  |  |  |  |
| 6 | x |  |  | x |  | x |  |  |  |  |
| 7 |  |  | x |  | x |  | x |  |  |  |
| 8 |  |  | x |  |  | x | x |  |  |  |
| 9 | x | x |  |  | x |  |  |  |  |  |
| ≥ 10 |  |  |  |  |  | x |  | x | x |  |

**DEEL 5. ALGEHELE BEOORDELING**

Als participanten alle conflicten hebben beoordeeld, wordt hen gevraagd om hun dag van gisteren te beoordelen. Het is hierbij de bedoeling dat ze naar hun dag in het algemeen kijken, niet naar specifieke episodes. Deze vragen bevinden zich achteraan de bundel.

*Om te eindigen gaan we nog eens de dag die je hebt beschreven in zijn geheel overlopen. Beantwoord hiervoor deze vragen* ***[FORMULIER ALGEMENE VRAGEN]****, en denk daarbij aan gisteren in het algemeen.*

Laat patiënten zelf de waarden aanduiden op papier. Indien nodig, kan verdere mondelinge toelichting worden gegeven bij de te beoordelen vragen.

Identificatiecode: ___________________

**INTERVIEW**

**BEOORDELING CONFLICTEN**

**ALGEMENE DAGBEOORDELING**

Voor het invullen van deze vragenlijst is het noodzakelijk dat u de **tabel** waarin u de conflicten die u vandaag ervaren staan in genoteerd, bij u neemt.

Indien u vragen heeft, aarzel niet om ze te stellen aan de onderzoeker.

**TE BEOORDELEN CONFLICT**

**DEEL 1: CONFLICTBEOORDELING**

**Conflictcode**: …………………………

Beantwoord nu onderstaande vragen die betrekking hebben op deze activiteiten en het conflict tussen deze activiteiten. *Omcirkel steeds het best passende cijfer.*

1. Hoe **sterk** ervoer je het conflict?

| 0  helemaal niet sterk | 1 | 2 | 3  neutraal | 4 | 5 | 6  heel erg sterk |
| --- | --- | --- | --- | --- | --- | --- |
|  |  |  |  |  |  |  |

1. **Beantwoord volgende vragen** die betrekking hebben op het conflict tussen je activiteiten.

|  | Helemaal niet |  |  | Neu-traal |  |  | Helemaal wel |
| --- | --- | --- | --- | --- | --- | --- | --- |
| Ik heb me **zorgen** gemaakt tijdens dit conflict | 0 | 1 | 2 | 3 | 4 | 5 | 6 |
| Ik heb me **zorgen** gemaakt over **pijn** tijdens dit conflict | 0 | 1 | 2 | 3 | 4 | 5 | 6 |
| Ik voelde me **gestresseerd** door dit conflict | 0 | 1 | 2 | 3 | 4 | 5 | 6 |
| Ik had nood aan steun tijdens dit conflict | 0 | 1 | 2 | 3 | 4 | 5 | 6 |

1. Hoe **moeilijk** vond je het om dit conflict **op te lossen**?

| 0  helemaal niet moeilijk | 1 | 2 | 3  neutraal | 4 | 5 | 6  heel erg moeilijk |
| --- | --- | --- | --- | --- | --- | --- |

1. Hoe **tevreden** ben je met de oplossing van dit conflict?

| 0  helemaal niet tevreden | 1 | 2 | 3  neutraal | 4 | 5 | 6  heel erg tevreden |
| --- | --- | --- | --- | --- | --- | --- |

1. In welke mate bent u het **eens** met volgende uitspraken? **Tijdens dit conflict heb ik me … gevoeld**. *Omcirkel het getal tussen 0 en 6 dat het beste beschrijft in welke mate u het eens bent met de uitspraak.*

|  | **Helemaal niet akkoord** |  | **Noch akkoord,**  **noch niet akkoord** | | |  | **Helemaal akkoord** |
| --- | --- | --- | --- | --- | --- | --- | --- |
|  | **0** | **1** | **2** | **3** | **4** | **5** | **6** |
|  |  |  |  |  |  |  |  |
| Blij | 0 | 1 | 2 | 3 | 4 | 5 | 6 |
| Enthousiast | 0 | 1 | 2 | 3 | 4 | 5 | 6 |
| Ontspannen | 0 | 1 | 2 | 3 | 4 | 5 | 6 |
| Triest | 0 | 1 | 2 | 3 | 4 | 5 | 6 |
| Zenuwachtig | 0 | 1 | 2 | 3 | 4 | 5 | 6 |
| Geïrriteerd | 0 | 1 | 2 | 3 | 4 | 5 | 6 |
| Kwaad | 0 | 1 | 2 | 3 | 4 | 5 | 6 |
| Bang | 0 | 1 | 2 | 3 | 4 | 5 | 6 |
| Machteloos | 0 | 1 | 2 | 3 | 4 | 5 | 6 |
| Gefrustreerd | 0 | 1 | 2 | 3 | 4 | 5 | 6 |
| Hulpeloos | 0 | 1 | 2 | 3 | 4 | 5 | 6 |
|  |  |  |  |  |  |  |  |

**TE BEOORDELEN CONFLICT**

**Conflictcode**: …………………………

Beantwoord nu onderstaande vragen die betrekking hebben op deze activiteiten en het conflict tussen deze activiteiten. *Omcirkel steeds het best passende cijfer.*

1. Hoe **sterk** ervoer je het conflict?

| 0  helemaal niet sterk | 1 | 2 | 3  neutraal | 4 | 5 | 6  heel erg sterk |
| --- | --- | --- | --- | --- | --- | --- |
|  |  |  |  |  |  |  |

1. **Beantwoord volgende vragen** die betrekking hebben op het conflict tussen je activiteiten.

|  | Helemaal niet |  |  | Neu-traal |  |  | Helemaal wel |
| --- | --- | --- | --- | --- | --- | --- | --- |
| Ik heb me **zorgen** gemaakt tijdens dit conflict | 0 | 1 | 2 | 3 | 4 | 5 | 6 |
| Ik heb me **zorgen** gemaakt over **pijn** tijdens dit conflict | 0 | 1 | 2 | 3 | 4 | 5 | 6 |
| Ik voelde me **gestresseerd** door dit conflict | 0 | 1 | 2 | 3 | 4 | 5 | 6 |
| Ik had nood aan steun tijdens dit conflict | 0 | 1 | 2 | 3 | 4 | 5 | 6 |
|  |  |  |  |  |  |  |  |

1. Hoe **moeilijk** vond je het om dit conflict **op te lossen**?

| 0  helemaal niet moeilijk | 1 | 2 | 3  neutraal | 4 | 5 | 6  heel erg moeilijk |
| --- | --- | --- | --- | --- | --- | --- |

1. Hoe **tevreden** ben je met de oplossing van dit conflict?

| 0  helemaal niet tevreden | 1 | 2 | 3  neutraal | 4 | 5 | 6  heel erg tevreden |
| --- | --- | --- | --- | --- | --- | --- |

1. In welke mate bent u het **eens** met volgende uitspraken? **Tijdens dit conflict heb ik me … gevoeld**. *Omcirkel het getal tussen 0 en 6 dat het beste beschrijft in welke mate u het eens bent met de uitspraak.*

|  | **Helemaal niet akkoord** |  | **Noch akkoord,**  **noch niet akkoord** | | |  | **Helemaal akkoord** |
| --- | --- | --- | --- | --- | --- | --- | --- |
|  | **0** | **1** | **2** | **3** | **4** | **5** | **6** |
|  |  |  |  |  |  |  |  |
| Blij | 0 | 1 | 2 | 3 | 4 | 5 | 6 |
| Enthousiast | 0 | 1 | 2 | 3 | 4 | 5 | 6 |
| Ontspannen | 0 | 1 | 2 | 3 | 4 | 5 | 6 |
| Triest | 0 | 1 | 2 | 3 | 4 | 5 | 6 |
| Zenuwachtig | 0 | 1 | 2 | 3 | 4 | 5 | 6 |
| Geïrriteerd | 0 | 1 | 2 | 3 | 4 | 5 | 6 |
| Kwaad | 0 | 1 | 2 | 3 | 4 | 5 | 6 |
| Bang | 0 | 1 | 2 | 3 | 4 | 5 | 6 |
| Machteloos | 0 | 1 | 2 | 3 | 4 | 5 | 6 |
| Gefrustreerd | 0 | 1 | 2 | 3 | 4 | 5 | 6 |
| Hulpeloos | 0 | 1 | 2 | 3 | 4 | 5 | 6 |
|  |  |  |  |  |  |  |  |

**TE BEOORDELEN CONFLICT**

**Conflictcode**: …………………………

Beantwoord nu onderstaande vragen die betrekking hebben op deze activiteiten en het conflict tussen deze activiteiten. *Omcirkel steeds het best passende cijfer.*

1. Hoe **sterk** ervoer je het conflict?

| 0  helemaal niet sterk | 1 | 2 | 3  neutraal | 4 | 5 | 6  heel erg sterk |
| --- | --- | --- | --- | --- | --- | --- |
|  |  |  |  |  |  |  |

1. **Beantwoord volgende vragen** die betrekking hebben op het conflict tussen je activiteiten.

|  | Helemaal niet |  |  | Neu-traal |  |  | Helemaal wel |
| --- | --- | --- | --- | --- | --- | --- | --- |
| Ik heb me **zorgen** gemaakt tijdens dit conflict | 0 | 1 | 2 | 3 | 4 | 5 | 6 |
| Ik heb me **zorgen** gemaakt over **pijn** tijdens dit conflict | 0 | 1 | 2 | 3 | 4 | 5 | 6 |
| Ik voelde me **gestresseerd** door dit conflict | 0 | 1 | 2 | 3 | 4 | 5 | 6 |
| Ik had nood aan steun tijdens dit conflict | 0 | 1 | 2 | 3 | 4 | 5 | 6 |
|  |  |  |  |  |  |  |  |

1. Hoe **moeilijk** vond je het om dit conflict **op te lossen**?

| 0  helemaal niet moeilijk | 1 | 2 | 3  neutraal | 4 | 5 | 6  heel erg moeilijk |
| --- | --- | --- | --- | --- | --- | --- |

1. Hoe **tevreden** ben je met de oplossing van dit conflict?

| 0  helemaal niet tevreden | 1 | 2 | 3  neutraal | 4 | 5 | 6  heel erg tevreden |
| --- | --- | --- | --- | --- | --- | --- |

1. In welke mate bent u het **eens** met volgende uitspraken? **Tijdens dit conflict heb ik me … gevoeld**. *Omcirkel het getal tussen 0 en 6 dat het beste beschrijft in welke mate u het eens bent met de uitspraak.*

|  | **Helemaal niet akkoord** |  | **Noch akkoord,**  **noch niet akkoord** | | |  | **Helemaal akkoord** |
| --- | --- | --- | --- | --- | --- | --- | --- |
|  | **0** | **1** | **2** | **3** | **4** | **5** | **6** |
|  |  |  |  |  |  |  |  |
| Blij | 0 | 1 | 2 | 3 | 4 | 5 | 6 |
| Enthousiast | 0 | 1 | 2 | 3 | 4 | 5 | 6 |
| Ontspannen | 0 | 1 | 2 | 3 | 4 | 5 | 6 |
| Triest | 0 | 1 | 2 | 3 | 4 | 5 | 6 |
| Zenuwachtig | 0 | 1 | 2 | 3 | 4 | 5 | 6 |
| Geïrriteerd | 0 | 1 | 2 | 3 | 4 | 5 | 6 |
| Kwaad | 0 | 1 | 2 | 3 | 4 | 5 | 6 |
| Bang | 0 | 1 | 2 | 3 | 4 | 5 | 6 |
| Machteloos | 0 | 1 | 2 | 3 | 4 | 5 | 6 |
| Gefrustreerd | 0 | 1 | 2 | 3 | 4 | 5 | 6 |
| Hulpeloos | 0 | 1 | 2 | 3 | 4 | 5 | 6 |
|  |  |  |  |  |  |  |  |

In wat volgt, dient u uw dag van gisteren in het algemeen te beoordelen. Omcirkel hiertoe het antwoord van jouw keuze.

**DEEL 2: ALGEHELE BEOORDELING**

1. Vergeleken met andere dagen de voorbije maand, hoe **typisch** was gisteren?

*Indien het gisteren een weekdag was, gelieve enkel te vergelijken met andere weekdagen. Indien het gisteren weekend was, gelieve enkel te vergelijken met andere weekenddagen.*

| 0 | 1 | 2 | 3 | 4 | 5 | 6 | 7 | 8 | 9 | 10 |
| --- | --- | --- | --- | --- | --- | --- | --- | --- | --- | --- |
| helemaal niet typisch |  |  |  |  |  |  |  |  |  | heel erg typisch |

1. Vergeleken met andere dagen de voorbije maand, **was gisteren**…

*Indien het gisteren een weekdag was, gelieve enkel te vergelijken met andere weekdagen. Indien het gisteren weekend was, gelieve enkel te vergelijken met andere weekenddagen.*

| 0 | 1 | 2 | 3 | 4 | 5 | 6 | 7 | 8 | 9 | 10 |
| --- | --- | --- | --- | --- | --- | --- | --- | --- | --- | --- |
| veel slechter |  |  |  |  |  |  |  |  |  | veel beter |

1. In welke mate ben je **tevreden** over je dag gisteren?

| 0 | 1 | 2 | 3 | 4 | 5 | 6 | 7 | 8 | 9 | 10 |
| --- | --- | --- | --- | --- | --- | --- | --- | --- | --- | --- |
| Helemaal niet tevreden |  |  |  |  |  |  |  |  |  | heel erg tevreden |

1. In welke mate heb je gisteren **gedaan wat je gepland** had te doen?

| 0 | 1 | 2 | 3 | 4 | 5 | 6 | 7 | 8 | 9 | 10 |
| --- | --- | --- | --- | --- | --- | --- | --- | --- | --- | --- |
| helemaal niet |  |  |  |  |  |  |  |  |  | Helemaal wel |

1. In welke mate heb je gisteren voor jou **waardevolle activiteiten** nagestreefd?

| 0 | 1 | 2 | 3 | 4 | 5 | 6 | 7 | 8 | 9 | 10 |
| --- | --- | --- | --- | --- | --- | --- | --- | --- | --- | --- |
| helemaal niet |  |  |  |  |  |  |  |  |  | Helemaal  wel |

1. Hoe **vermoeid** was je gisteren gemiddeld genomen doorheen de dag?

| 0 | 1 | 2 | 3 | 4 | 5 | 6 | 7 | 8 | 9 | 10 |
| --- | --- | --- | --- | --- | --- | --- | --- | --- | --- | --- |
| niet vermoeid |  |  |  |  |  |  |  |  |  | heel erg vermoeid |

1. Hoeveel **pijn** ervoer je gisteren gemiddeld genomen doorheen de dag?

| 0 | 1 | 2 | 3 | 4 | 5 | 6 | 7 | 8 | 9 | 10 |
| --- | --- | --- | --- | --- | --- | --- | --- | --- | --- | --- |
| geen pijn |  |  |  |  |  |  |  |  |  | ergst denkbare pijn |

1. In welke mate heeft **vermoeidheid** je gisteren gemiddeld gezien **belemmerd** tijdens het uitvoeren van je activiteiten?

| 0 | 1 | 2 | 3 | 4 | 5 | 6 | 7 | 8 | 9 | 10 |
| --- | --- | --- | --- | --- | --- | --- | --- | --- | --- | --- |
| helemaal niet |  |  |  |  |  |  |  |  |  | heel sterk |

1. In welke mate heeft **pijn** je gisteren gemiddeld gezien **belemmerd** tijdens het uitvoeren van je activiteiten?

| 0 | 1 | 2 | 3 | 4 | 5 | 6 | 7 | 8 | 9 | 10 |
| --- | --- | --- | --- | --- | --- | --- | --- | --- | --- | --- |
| helemaal niet |  |  |  |  |  |  |  |  |  | heel sterk |

1. In welke mate ben je gisteren **bezig geweest met vermoeidheid**?

| 0 | 1 | 2 | 3 | 4 | 5 | 6 | 7 | 8 | 9 | 10 |
| --- | --- | --- | --- | --- | --- | --- | --- | --- | --- | --- |
| helemaal niet |  |  |  |  |  |  |  |  |  | heel sterk |

1. In welke mate ben je gisteren **bezig geweest met pijn**?

| 0 | 1 | 2 | 3 | 4 | 5 | 6 | 7 | 8 | 9 | 10 |
| --- | --- | --- | --- | --- | --- | --- | --- | --- | --- | --- |
| helemaal niet |  |  |  |  |  |  |  |  |  | heel sterk |

1. In welke mate ben je het eens met volgende uitspraken? Over het algemeen heb ik me **gisteren … gevoeld.** *Omcirkel het getal tussen 0 en 6 dat het beste beschrijft in welke mate je het eens bent met de uitspraak. Gebruik hiervoor onderstaande antwoordschaal.*

|  | **Helemaal niet akkoord** |  | **Noch akkoord,**  **noch niet akkoord** | | |  | **Helemaal akkoord** |
| --- | --- | --- | --- | --- | --- | --- | --- |
|  | **0** | **1** | **2** | **3** | **4** | **5** | **6** |
|  |  |  |  |  |  |  |  |
| Blij | 0 | 1 | 2 | 3 | 4 | 5 | 6 |
| Enthousiast | 0 | 1 | 2 | 3 | 4 | 5 | 6 |
| Ontspannen | 0 | 1 | 2 | 3 | 4 | 5 | 6 |
| Triest | 0 | 1 | 2 | 3 | 4 | 5 | 6 |
| Zenuwachtig | 0 | 1 | 2 | 3 | 4 | 5 | 6 |
| Geïrriteerd | 0 | 1 | 2 | 3 | 4 | 5 | 6 |
| Kwaad | 0 | 1 | 2 | 3 | 4 | 5 | 6 |
| Bang | 0 | 1 | 2 | 3 | 4 | 5 | 6 |
| Machteloos | 0 | 1 | 2 | 3 | 4 | 5 | 6 |
| Gefrustreerd | 0 | 1 | 2 | 3 | 4 | 5 | 6 |
| Hulpeloos | 0 | 1 | 2 | 3 | 4 | 5 | 6 |

|  | **Helemaal niet akkoord** |  | **Noch akkoord,**  **noch niet akkoord** | | |  | **Helemaal akkoord** |
| --- | --- | --- | --- | --- | --- | --- | --- |
|  | **0** | **1** | **2** | **3** | **4** | **5** | **6** |
| Zelfverzekerd | 0 | 1 | 2 | 3 | 4 | 5 | 6 |
| Geliefd | 0 | 1 | 2 | 3 | 4 | 5 | 6 |
| Alert | 0 | 1 | 2 | 3 | 4 | 5 | 6 |
| Optimistisch | 0 | 1 | 2 | 3 | 4 | 5 | 6 |
| Rusteloos | 0 | 1 | 2 | 3 | 4 | 5 | 6 |
| Vastberaden | 0 | 1 | 2 | 3 | 4 | 5 | 6 |
| Energiek | 0 | 1 | 2 | 3 | 4 | 5 | 6 |

1. In welke was je vandaag **gemotiveerd om dit interview** te doorlopen?

| 0 | 1 | 2 | 3 | 4 | 5 | 6 | 7 | 8 | 9 | 10 |
| --- | --- | --- | --- | --- | --- | --- | --- | --- | --- | --- |
| helemaal niet gemotiveerd |  |  |  |  |  |  |  |  |  | heel erg gemotiveerd |

**EVENT SAMPLING: PROTOCOL**

Geef deelnemers de volgende instructies:

*Aan dit interview is ook een ander deel gekoppeld. Wij zouden u willen vragen om gedurende* ***14 opeenvolgende dagen*** *een* ***dagboekje*** *bij te houden. Het is de bedoeling dat u in dit dagboek bijhoudt hoeveel conflicten u ervaart, wanneer en tussen welke activiteiten deze conflicten zich voordeden. Met conflict bedoelen we opnieuw dat er twijfel of besluiteloosheid optreedt of iemand nu de ene dan wel een andere activiteit zal gaan uitvoeren. Van belang is ook dat men niet onmiddellijk tot een oplossing komt, en de twijfel een poosje aanhoudt. Het conflict moet zich voordoen tussen twee voldoende verschillende activiteiten. Deze definitie kan u ook terugvinden vooraan in het dagboek. Het is dus de bedoeling dat, zodra je een conflict ervaart, u dit zo snel mogelijk noteert in het boekje en de andere informatie aanvult. Is tot nu toe alles duidelijk?*

Beantwoord eventuele vragen. Leg vervolgens de vragenlijst uit die ze op het einde van de dag moeten invullen. Neem een papieren exemplaar erbij om alles te verduidelijken.

*Op het einde van elk van die 14 dagen zouden we u willen vragen omstreeks bedtijd een vragenlijst in te vullen. In deze vragenlijst vragen we u bijvoorbeeld aan te geven hoeveel conflicten u hebt ervaren, de onmiddellijk achterliggende doelen te classificeren voor beide activiteiten voor al uw ervaren conflicten, zoals we ook in het interview hebben gedaan, en verder nog enkele algemene vragen te beantwoorden over uw conflicten en uw dag in het algemeen. Dit zou ongeveer 15 minuten van uw tijd in beslag nemen. Ik wil nog eens extra benadrukken dat ook als u geen conflicten hebt ervaren, u de vragenlijst dient te vervolledigen. Is alles duidelijk?*

Beantwoord eventuele vragen. Leg uit:

*Om u te kunnen contacteren over het verloop van het dagboek na 7 dagen en na 14 dagen, alsook voor het versturen van een dagelijkse herinnering aan het invullen van de vragen, hebben wij uw GSM nummer nodig. Zou u mij kunnen zeggen wat uw GSM nummer is?*

Noteer het GSM nummer in het bestand dagboek data.

*De vragenlijsten behorend bij het dagboek worden bij voorkeur elektronisch ingevuld. Beschikt u over de mogelijkheid om het dagboek elektronisch in te vullen?* [vraag naar e-mailadres] *U zult, net zoals voor de algemene vragenlijsten een e-mail krijgen met daar in uw log-in gegevens en de link naar de vragenlijst. Dit zal 14 opeenvolgende dagen dezelfde login en dezelfde link blijven. Hou deze e-mail dus heel goed bij.*

Participanten die niet beschikken over een computer, krijgen zij de vragenlijsten mee op papier (14 bundels).

Vervolg met:

*We zouden u willen vragen om het ingevulde dagboek zo snel als mogelijk na afloop terug te sturen met de voorgefrankeerde enveloppe. [Indien participanten de vragenlijsten op papier invullen, wordt hen eveneens gevraagd om deze vragenlijsten zo snel als mogelijk, liefst de dag nadien, op de bus te doen]. Indien u het dagboek en de vragenlijsten minstens 10 opeenvolgende dagen invult, en op voorwaarde dat we u hebben kunnen bereiken voor de tussentijdse evaluatie van het dagboek en u het ingevulde dagboek (en de vragenlijsten) hebt teruggestuurd met de voorgefrankeerde enveloppe, krijgt u een additionele vergoeding van 20 euro die zal worden overgeschreven op uw bankrekening. Onder deelnemers die het dagboek 14 opeenvolgende dagen invullen, verloten we enkele FNAC-waardebonnen van 50 euro.*

Geef alle deelnemers het nodige materiaal (dagboek, vragenlijsten,…) mee en bedank hen voor hun medewerking.

1. Nathalie Claes (N.C.), Nele Decoene (N.D.), Eveline Demeulemeester (E.D.), and Julie Mengé (J.M.) [↑](#footnote-ref-1)
